# Event-Related Potential Evidence for Two Functionally Dissociable Sources of Semantic Effects in the Attentional Blink

**DOI:** 10.1371/journal.pone.0049099

**Published:** 2012-11-26

**Authors:** Francesca Peressotti, Francesca Pesciarelli, Claudio Mulatti, Roberto Dell'Acqua

**Affiliations:** 1 Department of Developmental and Social Psychology, University of Padova, Padova, Italy; 2 Department of Biomedical Sciences, University of Modena-Reggio Emilia, Modena, Italy; 3 Centre for Cognitive and Brain Science, University of Padova, Padova, Italy; CSIC-Univ Miguel Hernandez, Spain

## Abstract

Three target words (T1, T2, and T3) were embedded in a rapid serial visual presentation (RSVP) stream of non-word distractors, and participants were required to report the targets at the end of each RSVP stream. T2 and T3 were semantically related words in half of the RSVP streams, and semantically unrelated words in the other half of the RSVP streams. Using an identical design, a recent study reported distinct reflections of the T2–T3 semantic relationship on the P2 and N400 components of event-related potentials (ERPs) time-locked to T3, suggesting an early, automatic, source of P2 semantic effects and a late, controlled, source of N400 semantic effects. Here, P2 and N400 semantic effects were examined by manipulating list-wide context. Relative to participants performing in a semantically unbiased context, participants over-exposed to filler RSVP streams always including semantically related T2/T3 words reported a *dilution* of T3-locked P2 semantic effects and a *magnification* of T3-locked N400 semantic effects. Opposite effects on P2 and N400 ERP components of list-wide semantic context are discussed in relation to recent proposals on the representational status of RSVP targets at processing stages prior to consolidation in visual short-term memory.

## Introduction

Unconscious information processing has attracted the interest of researchers from the very early days of scientific psychology and even nowadays the nature of the mechanisms that mediate the influence of unconscious cognition is a highly debated and controversial issue. In the vast majority of cases, cognitive studies of unconscious cognition have made use of techniques devised to prevent conscious access to one visual stimulus, traditionally referred to as *prime*, and to probe its influence on the processing of a different, clearly visible and consciously perceived stimulus, traditionally referred to as *target*.

Recent theorization in this field has highlighted a critical subdivision of these techniques according to the way in which conscious access to prime stimuli is limited [1]. With one class of techniques, conscious perception of the prime is data-limited, often resorting to extremely brief prime exposures in conjunction with various forms of visual masking displayed in the close temporal surroundings of the prime. The masked priming paradigm is prototypical in this class. With another class of techniques, conscious perception of the prime (or target, depending on variants) is resource-limited by locking out attention mechanisms necessary for conscious perception (e.g., consolidation: [2]–[3]; tokenization: [4]–[5]). Prototypical in this class is the rapid serial visual presentation (RSVP) paradigm, in which primes and targets are embedded in streams of spatially overlapping distractors, and displayed at varying stimulus onset asynchronies (SOAs). When the SOA between prime and target is shorter than 500 ms, target identification is often precluded, owing to an attentional blink (AB [6]). RSVP stimuli are displayed at frequencies usually varying between 8 to 10 Hz, implying that prime exposure duration is less critical a factor to limit conscious perception using RSVP than the masked priming technique, e.g., [7].

Though often used to investigate distinct aspects of the functional and neural processing architecture, behavioral and electrophysiological results in the masked priming and RSVP/AB fields have separately converged on a set of assumptions concerning the representational status of unconscious visual stimuli. One such assumption is that unconscious stimuli access post-sensory stages of processing very rapidly [7], [8], [9], [10], [11], [12], [13], [14], [15], see [16], [17], for reviews in the AB field.

Furthermore, both fields have contributed evidence imposing a revisitation of the theoretical link between unconscious perception and automatic processing. Contrary to former proposals of unconscious stimuli as naturally bound to automatic processing (i.e., ballistic, of unlimited band-width or parallel, and uninfluenced by subjective control; e.g., [18], [19]), effects of unconscious stimuli using both masked priming and RSVP paradigms have been shown to be modulated by top-down, strategy-inducing, factors.

Temporal predictability is one such factor. Using masked priming, semantic priming has been shown to be abolished when participants are deprived of information on the timing of occurrence of either prime or target, [20]; see also [21] for similar evidence and conclusions. Analogously, AB effects have been shown to be strongly diminished when participants are informed verbally, or cued visually, about the temporal lag between two forthcoming sequential targets embedded in a RSVP stream of distractors [22], [23].

Task-set is another factor, whose influence on unconscious perception has been documented by monitoring a component of the event-related potential (ERP) characterized by its well-established susceptibility to semantic modulations, namely, N400 (i.e., an increase in negativity usually unfolding over a 300–500 ms time-window elicited by targets presented within semantically incongruent contexts, e.g., [24], [25]). Using masked priming, Kiefer and Martens [26] had prime/target words preceded by task-inducing words requiring, in one condition, a perceptual judgment, and a semantic judgment in a different condition. Unconscious N400 semantic priming effects were observed following a semantic judgment, and were nil following a perceptual judgment. Using the RSVP technique, Vachon and colleagues [27], [28] exposed participants to a clearly visible, to-be-memorized, prime word prior to the beginning of RSVP streams of non-words embedding two target words (T1 and T2), displayed at varying SOAs. When the task on T1 required a perceptual judgment, a blinked (missed) T2 at short T1–T2 SOA did not elicit N400 semantic priming effects. N400 semantic effects were however fully reinstated when the task on T1 required a semantic judgment (see [29] for similar findings).

Bodner and Masson [30], [31] reported findings using masked priming which are suggestive of a further top-down factor influencing unconscious processing. These authors manipulated list-wide context by systematically varying the relative proportion of semantically related versus unrelated prime/target words included in separate blocks of trials. Prior research using visible primes had indicated that priming effects tend to increase as the relative proportion of semantically related pairs in a trial block is increased [32], [33], [34], [35]. Bodner and Masson [30], [31] showed an analogous pattern varying the relative proportion of masked prime/target words. Based on this finding, the authors argued that, like with visible primes, masked priming effects are bound to the covert generation of memory traces for prime events which are subsequently recovered to subserve target processing, e.g., [36].

Curiously, the influence of list-wide context effects on the processing of unconscious stimuli has never been tested using the RSVP technique. Such test is the scope of the present investigation. The starting point was the study of Pesciarelli, Kutas, Dell'Acqua, Peressotti, Job, and Urbach [37], who displayed three target words (T1, T2, and T3) sequentially in RSVP streams composed of interleaving non-word distractors. T1 served the purpose to elicit an AB limiting conscious report of T2 on a proportion of trials. T3 words were displayed consistently outside the AB time-window. Participants had to report, at the end of each trial, the identity of the three target words. The results showed two distinct T3-locked ERP components sensitive to semantic modulations, namely, a component recorded in a 150–250 ms time-window (i.e., in the P2 range) whose positivity was amplified for related T2/T3 words, followed by classic N400 semantic priming effects. N400 semantic effects were taken to reflect integration of T3 within the semantic context elicited by T2 (e.g., [38], [39], [40], [41], [42], [43]; see [44] for a recent review and empirical evidence supporting this view; but see [45] for an alternative perspective). Earlier P2 semantic effects were taken to reflect fast propagation of bottom up volleys of semantic information ignited by T3 onset, (e.g., [46], [47], [48]). To implement a manipulation of list-wide context in the present study, *standard* RSVP/AB trials identical to those used by Pesciarelli et al. [37] were intermixed with *filler* RSVP trials in which prime (T2) and target (T3) words were unmasked and clearly visible, via omission of leading non-word distractors. In one condition, filler RSVP trials always included semantically related T2/T3 words, and in the other condition filler RSVP trials always included semantically unrelated T2/T3 words. The two conditions were administered to two different groups of participants. List-wide context effects were compared between the two groups of participants by considering standard RSVP trials only, namely, those RSVP trials that were in common between the two groups.

The manipulation of list-wide semantic context was included in the present design in order to disentangle two opposite hypotheses about the functional interplay between P2 and N400 semantic modulations reported by Pesciarelli et al. [37].

One hypothesis is that semantic effects on P2 were mere precursors of N400 semantic effects. On this hypothesis, participants exposed to filler RSVP trials always including semantically related T2/T3 should report a magnification of N400 semantic priming effects in standard RSVP trials. This would be so because N400 is held to index a semantic mismatch between T3 and semantic context established by a consciously perceived T2. If the coherence of the semantic context were increased by filler RSVP streams always including semantically related T2/T3, then the magnitude of an ERP response to a semantic mismatch between T2 and T3 embedded in standard RSVP trials should also increase, bringing about an amplification of N400 semantic effects in standard RSVP trials *vis-a-vis* identical trials accompanied by fillers always including semantically unrelated T2/T3. P2 semantic effects should parallel the expected result of a magnification of N400 semantic priming effects for participants exposed to filler RSVP trials always including semantically related T2/T3. As for N400, P2 semantic effects in standard RSVP trials should also increase when the coherence of the semantic context is increased by intermixing standard RSVP trials and filler RSVP trials always including semantically related T2/T3. Behaviorally, the results should adhere to this hypothetical ERP picture, and be compatible with prior indications of the independence of semantic processing of T2/T3 from a T1-elicited AB, as well as with the cited findings of Bodner and Masson [30], [31]. Priming effects on T3 report should be detected in standard RSVP trials whether or not T2 would be reported. In addition, priming effects on T3 should be amplified in standard RSVP trials when accompanied by filler RSVPs consistently including semantically related T2/T3.

A different hypothesis is that P2 and N400 semantic effects reflected functionally distinct processing stages, with N400 effects permeable to fillers-driven expectation about the occurrence of a semantic association between T2 and T3, and earlier P2 effects reflecting an early, automatic, response to T3 by a T2-preactivated semantic network. On this different hypothesis, list-wide context effects should modulate P2 and N400 semantic reflections differently, in line with studies suggesting that over-stimulating a semantic network via the repeated presentation of concepts sharing overlapping features can cause a phenomenon termed *satiation*, usually observed in the form of a progressive semantic responsiveness reduction – or habituation – as the proportion of semantically overlapping stimuli is increased (e.g., [49], [50]). Note that, if this were the case for participants exposed to filler RSVP trials always including semantically related T2/T3, the prediction concerning semantic effects on P2 and N400 semantic effects recorded in standard RSVP trials would diverge substantially, as the expected amplification of N400 semantic effects in standard RSVP trials should be accompanied by a concomitant attenuation of P2 semantic effects as the coherence of the semantic context is increased. Obverse effects on P2 and N400 components detected in standard RSVP trials would be incompatible with the idea of P2 semantic effects as precursors of N400 semantic effects. Rather, the results would support strongly a proposal of a different functional nature of the processing reflected in these components, at least under RSVP conditions. In standard RSVP trials accompanied by filler RSVPs including unrelated T2/T3, the behavioral results should replicate Pesciarelli et al. [37] results, as design and stimuli were identical between the present and that previous study. That is, priming effects should be evident on T3 report independently on whether or not T2 would be reported. The expected opposite semantic effects on P2 and N400 ERP components would likely surface in behavioral reports of T3 as nil semantic priming effects, for an increase in semantic coherence should result in an increment of semantic priming on T3, whereas an attenuation of semantic responsiveness owing to satiation should result in a decrement of semantic priming on T3. A note of caution, however, is in order with reference to the experimental paradigm used in the present study. Differently from what is typically observed with RSVP sequences including two target words, in which semantic priming effects in the AB are consistently reported (e.g., [27], [29]), semantic priming effects using the present three-target words RSVP variant are much less stable, with some studies reporting facilitatory semantic priming [37], [51], some others reporting no evidence of semantic priming at all [44], and one paradoxical case reporting a trend towards negative priming ensuing from semantically related prime/target stimuli [52]. We return on this inconsistency in the Discussion.

## Experiment

### Method

#### Ethical statement

The procedures have been approved by the Ethical Committee of the University of Padova.

#### Participants

Thirty-six students at the University of Padova (22 women), with an age ranging from 19 to 31 years (mean = 21 years) volunteered to participate in the present experiment. Participants were right-handed, native Italian speakers, without a history of neurological or mental disorders, with normal or corrected-to-normal visual acuity, and normal color vision. Written consent was obtained from each participant before the beginning of the experiment, as required by the Regulation of the Ethical Committee regarding cognitive/behavioral studies involving adult human participants.

#### Stimuli

The stimuli used for *standard* RSVP trials were one-hundred and twenty Italian 4-letters and 5-letters words selected from the VELI corpus [53] as T3 words. Each T3 word was paired two T2 words of identical length, a semantically related T2 word (e.g., It. OSSO – CANE; Eng. BONE – DOG) and a semantically unrelated T2 word (e.g., It. VELA – CANE; Eng. SAIL – DOG). T2 related and unrelated words were matched for frequency and orthographic neighborhood size. To minimize contamination by onset or orthographic priming effects, the initial letters of each T2–T3 pair were different and T2–T3 pairs had no more than two letters in common. T1 items were 120 words of the same length as T2 and T3. T1 words were semantically unrelated to both T2 and T3. Two separate lists of standard RSVP trials were generated by including identical T1 and T3 words. In each list, half of trials included T2–T3 semantically related words and the other half T2–T3 semantically unrelated words, such that each T3 word paired with a semantically related T2 word in one list was paired with a semantically unrelated T2 word in the other list. All participants were exposed to both lists, and the order of presentation of the lists was counterbalanced across participants.

The stimuli used for *filler* RSVP trials were 80 4-letters to 6-letters words selected from the same corpus as T3 words. Each T3 word was paired with two semantically related T2 words and two semantically unrelated T2 words, and 80 T1 words were selected to be semantically unrelated to both T2 and T3 words. Four separate lists of filler RSVP trials were generated by including identical T1 and T3 words. Two lists included 80 semantically related T2–T3 words, and two lists included 80 semantically unrelated T2–T3 words. Standard and filler RSVP trials included T1, T2, and T3 words and 19 non-words, each composed of a random sequence of 4 to 6 consonants. The structure of standard and filler RSVP trials was identical, except for the omission of the distractors in T1-1, T2-1, and T3-1 positions in filler RSVP trials. When displayed on screen, the maximum height and width of the stimuli were 0.5 and 3.2 degrees of visual angle. The background of the screen (i.e., a cathode-ray tube monitor controlled by a 686 Pentium CPU and E-prime software) was uniformly filled in gray. Distractors were displayed in black, T1 in green, and T2 and T3 in equiluminant red, each for a constant duration of 84 ms (0 ms inter-stimulus interval, or ISI). T1, T2, and T3 always occupied the fifth, eighth, and fifteenth positions in the sequence of distractors. The stimulus onset asynchrony (SOA) between T1 and T2 was thus 252 ms, and the SOA between T2 and T3 SOA was 588 ms.

#### Design and procedure

**Figure 1:**
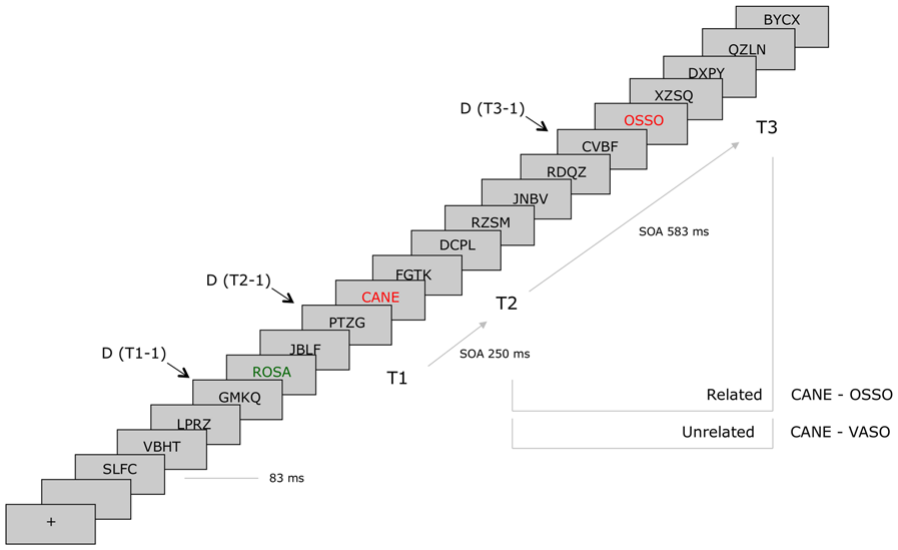
Temporal structure of a standard RSVP trial. Filler RSVP trials differed from standard RSVP trials for the replacement of distractors at positions T1-1, T2-1 and T3-1 with blank intervals of the same duration (84 ms). For half of the participants (high RP group), filler RSVP trials always included semantically related T2 and T3 words. For the other half of participants (low RP group), filler RSVP trials always included semantically unrelated T2 and T3 words.

An example of the sequence of events on one RSVP trial is illustrated in Figure 1.

Each trial began with three fixation crosses (‘+++’) at the center of the screen. Trials were self-administered using the spacebar on the keyboard connected to the same apparatus controlling stimuli presentation. A 700 ms blank interval following a spacebar press preceded the beginning of each RSVP stream. At the end of the RSVP stream, participants were instructed to type in, without time/speed pressure, T1, T2 and T3 in the same order as they appeared in the RSVP stream. Feedback was given at the end of each trial by replacing the ‘+’ in the position congruent with target order (from left to right, T1, T2, and T3) with a ‘−’ sign. The experiment consisted of 240 standard RSVP trials and 160 filler RSVP trials, randomly distributed in 10 blocks of 40 trials preceded by 1 block of 15 practice trials. Each participant was exposed to standard RSVP trials. Half of the participants were exposed to filler RSVP trials including only semantically unrelated T2 and T3 words, and the other half to filler RSVP trials including only semantically related T2 and T3 words.

#### EEG recording and analysis

Electroencephalographic (EEG) activity was recorded at a 250 Hz sampling rate from 19 tin electrodes held in place at standard 10/20 positions including Fp1, Fp2, Fz, F3, F4, F7, F8, C3, C4, Cz, P3, P4, Pz, O1, O2, T3, T4, P7, and P8 sites. Electrooculogram (EOG) activity was recorded from electrodes placed at the outer canthus and below each eye. EEG and EOG activities were referenced online to the left mastoid, and re-referenced off-line to the right and left mastoids. Data were amplified and bandpass filtered at 0.01–100 Hz, keeping impedance at each electrode site below 5 KΩ. Trials with artifacts due to eye movements, excessive muscle activity or amplifier saturation were eliminated off-line before event-related potential (ERP) estimation, using a 100 ms pre-stimulus averaged activity as baseline. ERPs affected by eye blinks were corrected using the algorithm proposed by Gratton, Coles, and Donchin [54]. T3-locked ERPs were generated only for standard RSVP trials associated with correct reports of T1 and T3. T3-locked ERP amplitude in the P2 range was quantified as mean activity recorded at frontal (F3, Fz, F4), and central (C3, Cz, C4) scalp sites in a 200–300 ms time-window, and in the N400 range as mean activity recorded at central (C3, Cz, C4) and posterior (P3, Pz, P4, O1, O2) scalp sites in a 300–480 ms time-window. T3-locked ERP amplitude values were submitted to analysis of variance (ANOVA), whose results were Greenhouse-Geisser corrected for non-sphericity when appropriate.

### Results

Only data from 24 participants (16 women, mean age = 22) were retained in the following analyses. Data from 12 participants had to be discarded because less than ten trials per cell of the design were left after removal of artifacts and standard RSVP trials associated with incorrect report of T1 or T3. Behavioral analyses were carried out on data from standard and filler RSVP trials, these latter differing in the two groups of tested participants, with one group exposed to semantically related filler RSVP trials (hereafter, high relatedness proportion, RP, group), and the other group exposed to semantically unrelated filler RSVP trials (hereafter, low RP group). The ERP analyses considered only results from standard RSVP trials.

#### Behavior

On average, the mean proportion of correct responses to T1, T2, and T3 was .90 in filler RSVP trials. The mean proportion of correct responses to T1, T2, and T3 was .74, .39, and .68, respectively, in standard RSVP trials. Individual mean values were submitted to ANOVA considering target (T1, T2, or T3) and RSVP trial type (standard vs. filler) as within-participant factors. The ANOVA indicated significant main effects of target, *F*(2, 46) = 98.5, *p*<.0001, *η_p_^2^* = .81, of trial type, *F*(1, 23) = 280.9, *p*<.0001, *η_p_^2^* = .92, and a significant interaction between these factors, *F*(2, 46) = 123.9, *p*<.0001, *η_p_^2^* = .84. A separate analysis on filler RSVP trials showed that the proportion of correct responses did not differ across T1, T2, and T3 (*F*<1). Separate *t*-tests contrasting each combination of targets in standard RSVP trials showed a reduced proportion of correct responses to T2 and T3 relative to T1, *t*(23) = 13.5, *p*<.0001; *t*(23) = 2.8, *p*<.01, and a reduced proportion of correct responses to T2 relative to T3, *t*(23) = 13.0, *p*<.0001, consistent with a T1-triggered AB effect affecting T2.

**Figure 2:**
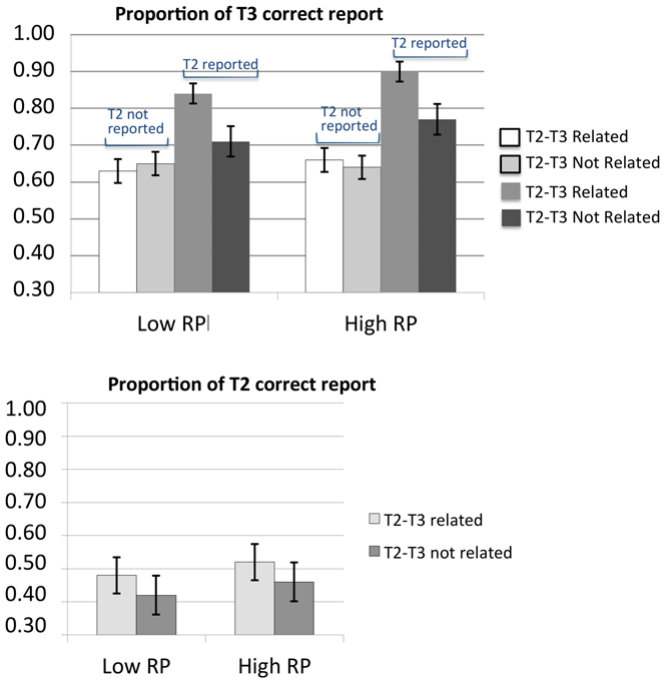
Behavioral results. Top panel: Proportion of correct responses to T3 (given T1 correctly reported) in standard RSVP trials plotted as a function of RP group (low vs. high), T2–T3 semantic relatedness (related vs. unrelated), and T2 status (missed vs. correctly reported). Bottom panel: Proportion of correct responses to T2 (given T1 correctly reported) in standard RSVP trials plotted as a function of RP group and T2–T3 semantic relatedness.

Standard RSVP trials were submitted to an additional ANOVA considering RP group (high vs. low) as a between-participant factor, and T2–T3 semantic relatedness (semantically related vs. unrelated) as a within-participant factor. The analyses were conducted separately for each target. A summary of the results is illustrated in Figure 2.

The ANOVA on the proportion of correct responses to T1 found no significant factor effects (*F*s<1). The ANOVAs on T2 and T3 were conducted on data from standard RSVP trials associated with a correct report of T1.

The ANOVA on the proportion of correct responses to T2 revealed a significant main effect of T2–T3 relatedness, *F*(1, 22) = 28.6, *p*<.0001, *η_p_^2^* = .57, indicating modest priming effects (relative to those observed on T3; see Figure 2) exhibited by both the high and low RP groups. No other factor or interaction was significant in this analysis (max *F* = 1.9; min *p*>.1). Priming effects on T2 by T3 are not unusual using this type of designs requiring memory maintenance and delayed report of targets, and are likely to reflect a bias on the part of participants to report and/or guess correctly a T2 semantically related to T3 on a small fraction of standard RSVP trials.

The ANOVA on the proportion of correct responses to T3 considered T2 status (missed vs. correctly reported) as an additional within-participant factor. The analysis revealed a significant main effects of T2 status, *F*(1, 22) = 64.4, *p*<.0001, *η_p_^2^* = .75, of T2–T3 relatedness, *F*(1, 22) = 26.6, *p*<.0001, *η_p_^2^* = .55, and a significant interaction between these factors, *F*(1, 22) = 20.5, *p*<.0001, *η_p_^2^* = .48. No other factor or interaction were significant in this analysis (all *F*s<1). As Figure 2 makes clear, semantic priming effects were detected on T3 only under conditions of correct T2 report. Behaviorally, this pattern was common to both high and low RP groups.

#### T3-locked ERPs: P2

Individual ERP amplitude values in the P2 range (see ‘EEG recording and analysis) were submitted to an ANOVA considering RP group (high vs. low) as between-participant factor, and T2 status (missed vs. correctly reported), T2–T3 relatedness and electrode location as within-participant factors.

**Figure 3:**
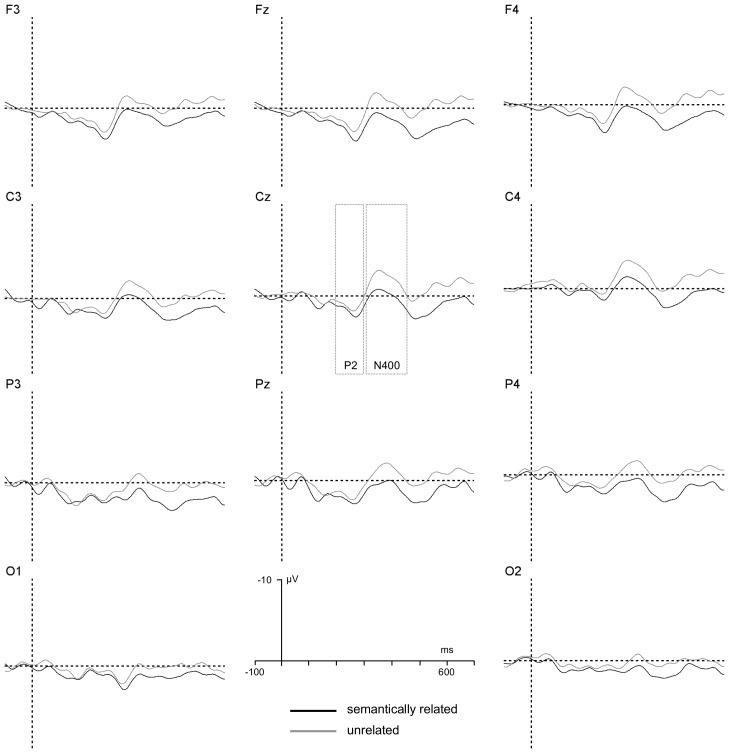
T2 reported, Low RP group. T3-locked grand-average ERPs generated by considering standard RSVP trials (given T1 and T3 correctly reported) performed by the low RP group for trials in which T2 was correctly reported. A graphical indication of the time-windows used for estimation of P2 and N400 components' amplitudes is reported at Cz.

**Figure 4:**
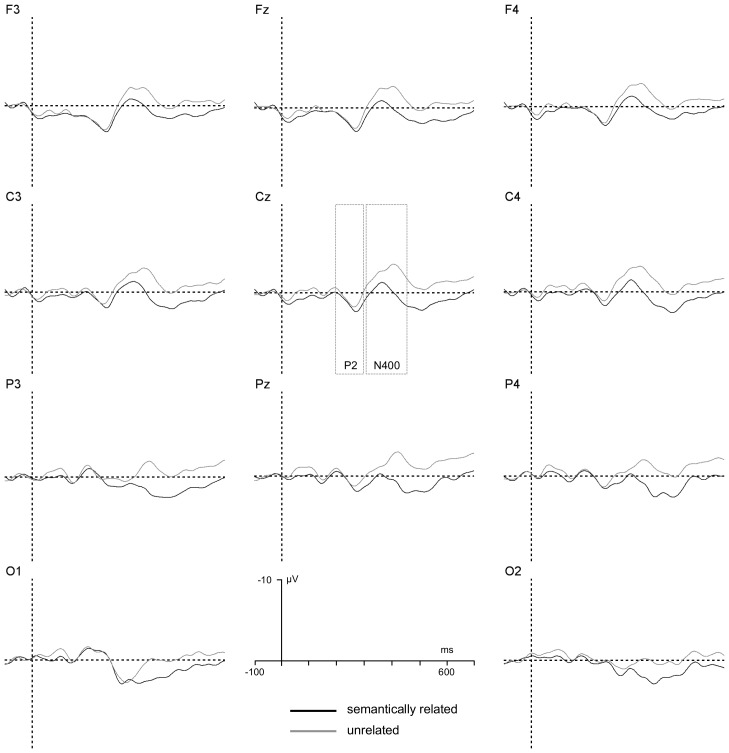
T2 reported, High RP group. T3-locked grand-average ERPs generated by considering standard RSVP trials (given T1 and T3 correctly reported) performed by the high RP group for trials in which T2 was correctly reported. A graphical indication of the time-windows used for estimation of P2 and N400 components' amplitudes is reported at Cz.

The ANOVA revealed two significant interactions, one between T2–T3 relatedness and T2 status, *F*(1, 22) = 4.3, *p*<.05, *η_p_^2^* = .15, suggesting that semantic effects were generally larger in trials in which T2 was reported relative to trials in which T2 was missed, and the other between T2–T3 relatedness and group, *F*(1, 22) = 4.7, *p*<.04, *η_p_^2^* = .18, suggesting that semantic effects on P2 amplitude were reliable only in the low RP group. Separate analyses on P2 amplitudes were conducted for standard RSVP trials broken down by T2 status. A summary of the results when T2 was correctly reported is illustrated in Figures 3 and 4.

**Figure 5:**
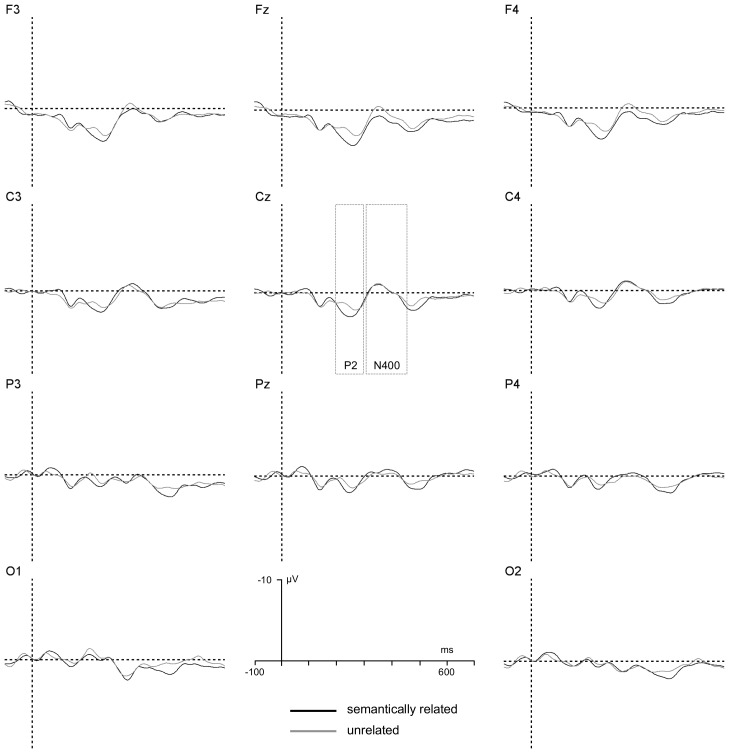
T2 not reported, Low RP group. T3-locked grand-average ERPs generated by considering standard RSVP trials (given T1 and T3 correctly reported) performed by the low RP group for trials in which T2 was missed. A graphical indication of the time-windows used for estimation of P2 and N400 components' amplitudes is reported at Cz.

**Figure 6:**
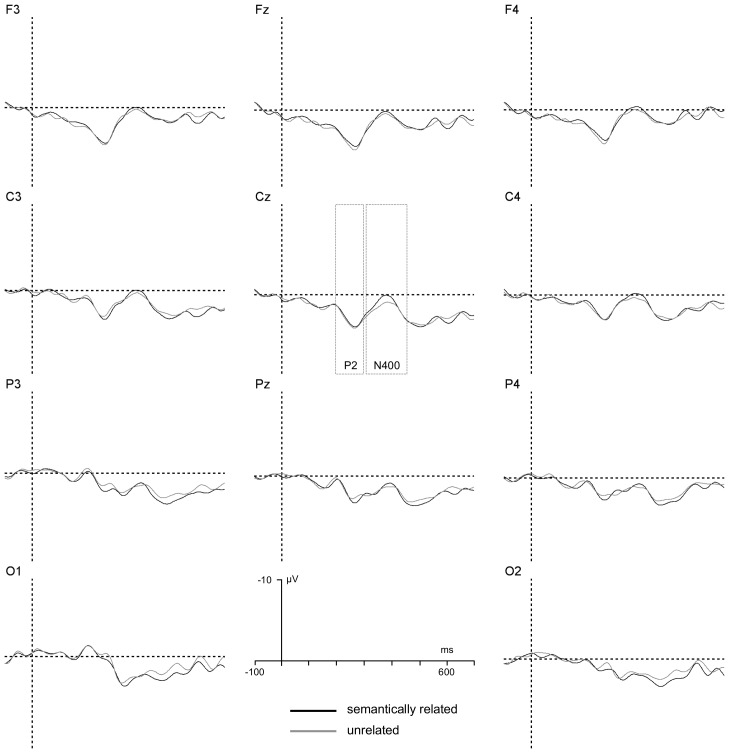
T2 not reported, High RP group. T3-locked grand-average ERPs generated by considering standard RSVP trials (given T1 and T3 correctly reported) performed by the high RP group for trials in which T2 was missed. A graphical indication of the time-windows used for estimation of P2 and N400 components' amplitudes is reported at Cz.

When T2 was correctly reported, the ANOVA revealed a significant interaction between T2–T3 relatedness and group, , *F*(1, 22) = 4.3, *p*<.05, *η_p_^2^* = .15. An additional analysis conducted on P2 amplitude values recorded on T2-correct standard RSVP trials indicated a significant main effect of T2–T3 relatedness for the low RP group, *F*(1, 11) = 4.9, *p*<.05, *η_p_^2^* = .2, but not for the high RP group, *F*<1. A summary of the results when T2 was missed is illustrated in Figures 5 and 6.

When T2 was missed, the ANOVA revealed significant main effects of T2–T3 relatedness, *F*(1, 22) = 3.2, p<.05, *η_p_^2^* = .15, of electrode location, *F*(5, 110) = 7.0, *p*<.007, *η_p_^2^* = .24, indicating a larger P2 amplitude at frontal electrode sites, and a significant interaction between T2–T3 relatedness and RP group, *F*(1, 22) = 4.8, *p*<.05, *η_p_^2^* = .18. An additional analysis conducted on P2 amplitude values recorded on T2-missed standard RSVP trials indicated a significant main effect of T2–T3 relatedness for the low RP group, *F*(1, 11) = 11.0, *p*<.005, *η_p_^2^* = .5, but not for the high RP group, *F*<1.

**Figure 7:**
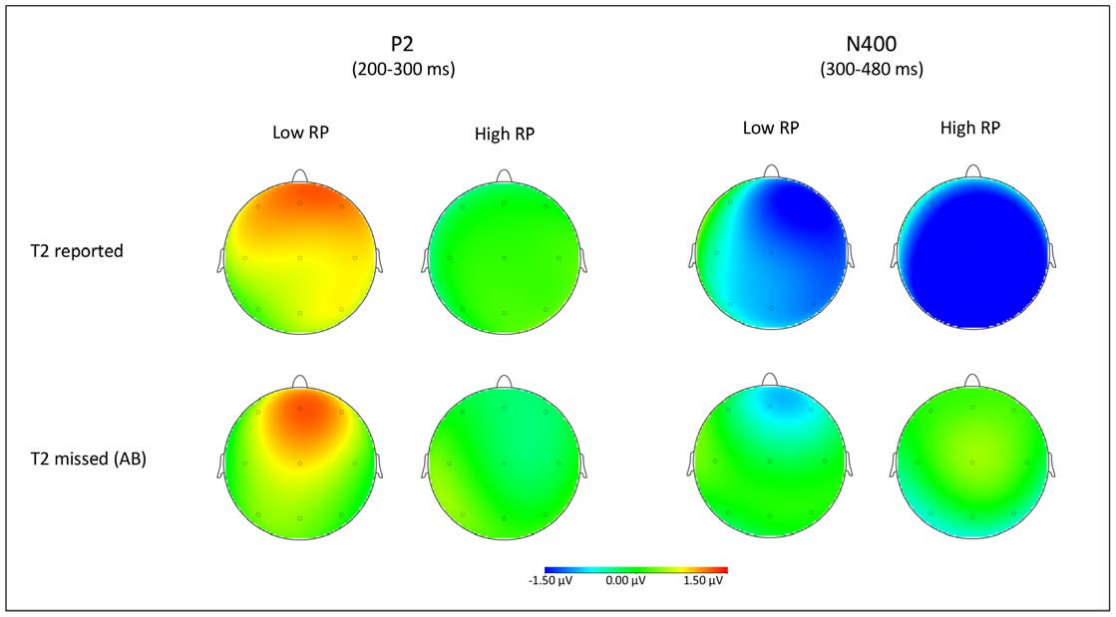
Difference wave scalp topographies. T2–T3 relatedness effect in the P2 (i.e., 200–300 ms) and N400 (i.e., 300–480) time-windows, as a function of RP group (high vs. low) and T2 status (correctly reported vs. missed).

To further explore the interactions obtained, net priming effects on T3-locked P2 amplitude values were calculated by subtracting ERPs recorded on standard RSVP trials including semantically unrelated T2–T3 targets from ERPs recorded on standard RSVP trials including semantically related T2–T3 ERPs. These difference ERPs are illustrated as scalp topographic maps in Figure 7 as a function of T2 status and RP group.

Individual difference P2 amplitude values were submitted to ANOVA, considering RP group (high vs. low) as between-participant factor and electrode location as within-participant factor. As can be seen from Figure 7, a reliable effect of group was observed on T2-missed and T2-correct standard RSVP trials, *F*(1, 22) = 4.4, *p*<.05, *η_p_^2^* = .16 and *F*(1, 22) = 4.8, *p*<.04, *η_p_^2^* = .17, respectively, reflecting non-nil P2 semantic effects for the low RP group only.

When the data from the different electrode sites were pooled, separate *t*-tests indicated that semantic effects on P2 amplitude were evident for the low RP group in both T2-correct and T2-missed standard RSVP trials, *t*(12) = 2.3, *p*<.04, and *t*(12) = 2.1, *p*<.05, respectively, while in the high RP group no evidence of semantic priming in the form of non-nil P2 difference activity was observed (Figure 7).

This pattern suggests that priming effects on T3 were manifest in T3-locked P2 amplitude increments that, albeit of different amplitude, were present both for T2-correct and T2-missed trials. Importantly, however, such priming effects were observed in standard RSVP trials performed by the low RP group, and were absent in standard RSVP trials performed by the high RP group.

#### T3-locked ERPs: N400

Individual ERP amplitude values in the N400 range (see ‘EEG recording and analysis) were submitted to ANOVA considering RP group (high vs. low) as between-participant factor, and T2 status (missed vs. correctly reported), T2–T3 relatedness, and electrode location as within-participant factors.

The ANOVA revealed two significant interactions, one between T2–T3 relatedness and T2 status, *F*(1, 22) = 4.6, *p*<.04, *η_p_^2^* = .17, and the other between T2–T3 relatedness and group, *F*(1, 22) = 5.5, *p*<.03, *η_p_^2^* = .2. The former interaction revealed the fact that N400 effects were observed only for T2 reported trials, the latter that these effects were larger for the high RP than for the low RP group. Separate analyses on N400 amplitudes were conducted for standard RSVP trials broken down by T2 status. A summary of the results when T2 was correctly reported is illustrated in Figures 3 and 4.

When T2 was correctly reported, the ANOVA indicated significant main effects of T2–T3 relatedness, *F*(1, 22) = 13.0, *p*<.002, *η_p_^2^* = .37, of electrode location, *F*(7, 154) = 7.9, *p*<.0001, *η_p_^2^* = .26, and a significant interaction between T2–T3 relatedness and RP group, *F*(1, 22) = 3.8, *p*<.05, *η_p_^2^* = .15, reflecting magnified classic, centro-parietal, N400 semantic priming effects for the high RP group relative to the low RP group.

Net priming effects on T3-locked N400 amplitude values were calculated by subtracting ERPs recorded on standard RSVP trials including semantically unrelated T2–T3 targets from ERPs recorded on standard RSVP trials including semantically related T2–T3 ERPs. These difference ERPs are illustrated as scalp topographic maps in Figure 7 as a function of T2 status and RP group. Individual difference N400 amplitude values were submitted to ANOVA, considering RP group (high vs. low) as a between-participant factor and electrode location as a within-participant factor. The analysis revealed a significant effect of group when T2 was reported, *F*(1, 22) = 4.9, *p*<.04, *η_p_^2^* = .18, but not when T2 was missed, reflecting larger N400 semantic effects for the high RP group when T2 was correctly reported, and nil N400 semantic effects when T2 was missed (Figure 5 and 6). When the data from the different electrode sites were pooled, separate one-pair *t*-tests indicated that semantic effects on N400 amplitude were significantly non-nil for the low and high RP groups on T2-correct trials, *t*(12) = 2.6, *p*<.03 and *t*(12) = 4.2, *p*<.001, respectively. On T2-missed trials, no significant effect of T2–T3 relatedness was detected, *t*s< = 1. As is evident in Figure 7, N400 semantic priming effects were apparent when T2 was correctly reported, and were absent when T2 was missed. This pattern held true for both the high RP and low RP groups.

## Discussion

The present investigation moved its steps from observing a general analogy between results produced using the masked priming and RSVP techniques when the influence of top-down factors on unconscious processing was considered. Previous work showed that this analogy was observed when manipulating temporal expectation, order predictability, and task-set using both techniques (e.g., [20], [22], [26], [27]). List-wide context was also shown to exert effects on unconscious processing using masked priming [30], [31], and we noticed that an analogous test has never been provided using the RSVP technique. To fill this gap, list-wide context was manipulated by administering to two distinct groups of participants an identical set of standard RSVP trials embedding semantically related and unrelated T2 and T3 words. Standard RSVP trials were randomly intermixed with filler RSVP trials that were generated by replacing pre-target distractors with blank intervals. This expedient was adopted to maximize the visibility of T1, T2, and T3 words in these trials. Filler RSVP trials administered to one group of participants, the low RP group, contained T2 and T3 words that were consistently semantically unrelated. Filler RSVP trials administered to a different group of participants, the high RP group, contained T2 and T3 words that were consistently semantically related. Filler RSVP trials were meant to elicit a semantic context characterized by a higher degree of coherence of the portion of semantic network stimulated by T2 and T3 concepts (for the high RP group) relative to a semantically unbiased context generated by non-overlapping T2 and T3 concepts (for the low RP group). Context effects in standard RSVP trials were monitored both behaviorally and electrophysiologically, focusing in this latter case on T3-locked ERP components that prior work [37] indicated as sensitive to semantic manipulations, namely, P2 and N400 components.

Behaviorally, the proportion of correct target words report, though showing clear AB effects on T2, did not reflect context effects on T3. Furthermore, semantic priming effects on T3 were detected in standard RSVP trials only when T2 was correctly reported. Smaller priming effects were also detected on T2, reflecting a bias to report semantically related words at a stage of retrieval of consolidated T3 words from visual short-term memory, and not at encoding stages during RSVP processing. Compatibly with this view, T2-locked ERP responses showed no effects of the semantic relation between T2 and T3 in standard RSVP trials.

Inconsistently with the predictions, null priming effects were reported for trials in which T2 was missed not only for the high RP group, but also for the low RP group. As already pointed out in the Introduction, previous evidence using a similar paradigm showed a quite inconsistent picture, with some studies reporting behavioral priming [37], [51] and some studies that did not [44], [52]. This suggests that offline (i.e., delayed) measures of performance in three-word RSVP paradigms may not be sensitive enough to capture the semantic priming effects that were instead fully fledged in ERPs. In fact, ERPs represent an more suitable tool to track – ms-to-ms – the effects of list-wide semantic context on the processing of unconscious stimulation at *encoding* stages. Behavioral estimates, in contrast, are prone to the influence of factors affecting *memory*, such as fading of the memory traces, report order confusion, and guessing, especially when one or more targets were missed owing to an AB effect. An additional limitation of behavioral estimates of priming effects in the present context would also arise from the relative duration of priming effects, which have been shown to be sometimes short-lived [55], [56]. Short-lived priming effects would terminate to exert their influence on behavior largely before a delayed response (usually made a few seconds after each RSVP stream ending) would be emitted. Moreover, the fact that at the behavioral level the priming effect obtained for trials in which T2 was reported was not modulated by filler type is inconsistent with what is generally found within the priming literature with visible primes, which shows enhanced priming effects when the majority of the prime-target word pairs are related [56]. As suggested above, this might be due to a problem of measure sensitivity, or to a ceiling effect, this latter limiting factor suggested by the high proportion of correct responses to T3 provided by participants in the high RP group, which exceeded the values of .9 in standard RSVP trials including semantically related T2/T3.

The electrophysiological results suggest both the T3-locked ERP components of interest were modulated by list wide context. N400 priming effects were of greater magnitude in the high RP group than in the low RP group on T2-correct standard RSVP trials (for similar findings, see [37], [44]). P2 priming effects were also modulated by context, though in a way opposite to N400. That is, both when T2 was consciously perceived and missed, P2 priming effects were detected in standard RSVP trials administered to the low RP group, but were reduced to nil in standard RSVP trials administered to the high RP group. The effect in the low RP group were larger when T2 was reported than when T2 was missed, consistently with the fact that semantic priming effects are usually larger for clearly visible primes than for masked primes [39], [57]. The pattern, therefore, is incompatible with the proposal of an analogous nature of semantic effects on P2 and N400 components, and compatible with the alternative view hypothesized in the Introduction that semantic effects on P2 and N400 ERP components are more likely functionally dissociable reflections of semantic processing of RSVP stimuli. To wit, relative to participants performing in a semantically unbiased context, participants over-exposed to filler RSVP streams always including semantically related T2/T3 words reported a *dilution* of T3-locked P2 semantic effects and a *magnification* of T3-locked N400 semantic effects.

The present ERP findings suggest an interpretation that corroborate and extend the proposal of a dual-nature of the processing reflected in T3-locked P2 and N400 components observed in RSVP processing. P2 semantic effects have a bottom-up origin, reflecting rapid propagation of T2-influenced semantic activation from short-range neural circuitries in visual extrastriate areas, likely including infero-temporal circuitries, to higher-level, likely pre-frontal, processing areas. The latency of P2 semantic effects is compatible with estimates of objects' processing that locate temporally the generation of such bottom-up volleys of semantic activity in a 120–200 ms time-window [46], [47], [48], [58], [59], [60], [61], [62], [63], [64]. Like in Pesciarelli et al. [37], evidence of T3-locked P2 priming effects was present for both blinked and reported T2s, compatibly with its proposed *structural* origin. Also, the fact that the effect was larger when T2 was reported supports the hypothesis that consciously perceived primes generate stronger bottom up spreading of activation effects than primes that are not consciously perceived.

Activity reflected in P2 semantic effects is susceptible to satiation, namely, a reduction of responsiveness to meaningful input whereby the portion of semantic network generating P2 responses is over-stimulated by increasing the proportion – on the time-scale of minutes – of semantically coherent T2 and T3 stimuli. Incidentally, in a recent AB work, effects analogous to satiation were found even under condition in which the rate (not proportion) of stimuli of a given alphanumeric class was increased, resulting in a reduced proportion of correct target responses when targets were clustered temporally relative to a condition in which they were separated by long intervals [65]. Although we find this idea highly plausible, it is frustrating that work corroborating this position is presently scant (see, however, the Introduction, and [49], [50]).

Congruently with work overviewed in the Introduction, the present results corroborate the idea that activity reflected in N400 semantic effects is permeable to top-down factors. Under conditions of repeated exposure of semantically coherent stimuli over the course of the experiment, one may imagine the payoff of anticipating a semantically coherent T3 following T2 may have been of secure appeal to participants, who likely adopted it as a general strategy in order to maximize the probability of a correct T3 report. This interpretation makes less surprising that N400 semantic effects were observed in T2-correct standard RSVP trial only, that is, when participants had a clear representation of the T2 word and its meaning. Obviously, a magnified N400 (mismatch) response had to be expected, as was indeed the case shown herein, when a semantically incongruent T3 followed a visible T2 when participants adopted the above anticipatory strategy. A note of clarification may be in order when comparing the present results with AB/ERP results described in prior work showing N400 semantic effects from missed targets (e.g., [27], [29]). The discrepancy is clearly only apparent, as the methods used in the present empirical context and in these prior studies diverge under a fundamental aspect. In three-target RSVP streams like those used in the present study, T1 served the purpose to produce an AB affecting T2 processing, yielding a certain proportion of unconscious T2, *prime*, words. Clearly visible T3, *target*, words were displayed consistently outside the AB time-window, and semantic priming effects were estimated both in the form of behavioral priming effects on T3 report accuracy, and in the form of T3-locked ERP waveforms' changes as a function of T2–T3 semantic relation. Vachon and Jolicœur [27] (see also [29]) used the expedient of showing a clearly visible *prime* stimulus generating a semantic context for an unlimited duration prior to each RSVP stream, and what was resource-limited via a T1-triggered AB perturbation was an unconscious T2, *target*, words.

N400 semantic effects have a top-down origin, reflecting T3 integration within a pre-established, T2-influenced, semantic context. There is ground to believe that latency and peculiarities of this component may be tied to cross-talking among anterior brain areas, including fronto- and dorso-lateral circuitries and their symmetric connections with the anterior parts of both temporal lobes (e.g., [41], [66]). The idea that the N400 reflects integrative processes is rooted in a number of studies that described target-locked, semantically driven N400 effects only with clearly visible primes (e.g., [39], [67]). However, alternative views postulate that N400 indexes facilitated access to lexical representations from long-term memory [24], [68] and, consistently with this interpretation, some studies showed significant N400 effects even for masked primes [21], [69], [70]. As highlighted by Holcomb et al. [67], however, the fact that the N400 is modulated by masked primes only suggest that the N400 is sensitive to the context generated by a masked stimulus and it does not necessarily exclude the possibility that the N400 indexes integration mechanisms.

It is not straightforward to forge a link between the present findings and prior work in which list-wide semantic context was varied using masked priming [30], [31]. Over and above the general consistency of the present findings with the idea that list-wide context does exert an effect in RSVP processing, one should note Bodner and Masson [30], [31] varied the proportion of *masked* prime/target words. In the present study, filler RSVP trials displayed prime/target words that were *clearly visible*. Furthermore, Bodner's and Masson [30], [31] manipulation was parametrically controlled, whereas this type of control can hardly be achieved using RSVP designs, owing to the notorious inter-individual variability in AB susceptibility (e.g., [71]), as well as to spontaneous fluctuations in preparation state which are the focus of the most recent electrophysiological explorations in the AB field [72], [73]. At present, therefore, the manipulation implemented in the masking priming paradigm may not find a straightforward application in the RSVP/AB domain of investigation, making a direct, conceptual and/or parametric, comparison between Bodner and Masson's and the present results premature.

Perhaps, this may be one of those cases where either technique (RSVP and masked priming) discloses its inherent limitations. Pros aside, one con of the masked priming technique is the impossibility to monitor the visibility of primes on a per-trial basis [74]. This turns to a pro in RSVP, where trials can be partitioned just based on whether an item (like T2 in the present case) has been successfully reported or not. In this perspective, it is interesting to note how elusive is the representational status of missed targets in RSVP to conscious report, as though missed targets were really ‘invisible’ to participants. Sergent and Dehaene [75], (see also [76]) have shown that information about missed and reported targets is dichotomously distributed, and do not generate a graded continuum contemplating ‘partial information recovery’ (e.g., “I really cannot say what word I saw, but I am pretty sure the initial syllable was DA”) from a degraded (missed) targets. Quite in contrast, participants' reports of missed targets is incredibly poor, as though participants did not retain the least information about such targets. Pattern masking, as usually employed in masked priming, is notoriously less effective in this vein, leaving open the possibility to prime/target integration phenomena, which are less reassuring in point of primes' ‘invisibility’ (e.g., [77], [78], [79]). Our argument here is not one aimed at disproving Bodner and Masson's [30], [31] findings and conclusions. Rather, the present should be taken as a warm invitation to caution when endeavoring to compare results from the RSVP and masked priming fields of investigation, an effort that cannot prescind from considering the macroscopic differences at the functional and neural level [1] as well as the apparently microscopic deviations, that cannot be easily obviated and structurally implemented in the RSVP and masked priming paradigms.

## Conclusion

The results here reported show that the semantic priming in the AB is the result of two independent processes, one evident as an early modulation of the ERPs, 200 ms after target presentation, and reflecting bottom-up semantic activation triggered by the presentation of a target word. The other process manifested itself as later N400 amplitude modulations, likely reflecting context integration processes. Both processes were shown to be permeable to list-wide context. The early effect, which was independent of prime visibility, is present when the system is not biased in the processing of the RSVP trial and it disappears in conditions in which the task induces participants to generate expectations on the semantic relationship between the target words they have to report. The late effect, which was observed only when the prime was consciously reported, is magnified in such conditions. The dissociation between early and late semantic effects is inspiring for models of word recognition, as it suggests that a subset of processing stages up to and including an early stage of semantic processing are ballistically activated upon presentation of linguistic stimuli. The present results suggest also that these automatic stages are partially independent from those required to generate a word representation available for conscious report.
